# ^31^P‐MRS of the Human Heart at 7 T With an Integrated Whole‐Body ^31^P Radiofrequency Transmit Coil

**DOI:** 10.1002/nbm.70248

**Published:** 2026-02-19

**Authors:** Mark W. J. M. Gosselink, Martijn Froeling, Kathy Verkerk, Dennis W. J. Klomp, Adrianus J. Bakermans, Jeanine J. Prompers

**Affiliations:** ^1^ Department of Radiology University Medical Center Utrecht Utrecht the Netherlands; ^2^ Department of Radiology and Nuclear Medicine Amsterdam University Medical Center, University of Amsterdam Amsterdam the Netherlands; ^3^ Department of Human Biology NUTRIM Institute of Nutrition and Translational Research in Metabolism, Maastricht University Medical Center+ Maastricht the Netherlands; ^4^ Department of Radiology and Nuclear Medicine Maastricht University Medical Center+ Maastricht the Netherlands

**Keywords:** ^31^P‐MRSI, birdcage RF coil, heart failure, high‐energy phosphate metabolism, reproducibility, ultra‐high‐field MRI, X‐nuclei

## Abstract

Phosphorus‐31 magnetic resonance spectroscopy (^31^P‐MRS) can be used to characterize the steady‐state in vivo myocardial energy status via the phosphocreatine (PCr) over adenosine triphosphate (ATP) tissue concentration ratio. Although used in many studies, this modality suffers from low sensitivity and poor measurement precision. This work reports on the acquisition of ^31^P‐MR spectra of the in vivo human heart using a 7 T MR system equipped with an integrated whole‐body RF transmit coil for ^31^P spin excitation. Eight volunteers (male/female, *n* = 4/4) underwent ^31^P‐MRS at 7 T twice, using a low‐flip angle, short‐repetition time 3D ^31^P‐MR spectroscopic imaging sequence with a 20‐mm isotropic resolution and a 21‐min acquisition time. A 16‐channel ^31^P receive array was used for signal reception. Myocardial voxels were identified on transversal cine proton MR images. Signals of PCr, phosphodiesters (PDE), and inorganic phosphate (P_i_) were quantified relative to ATP and corrected for partial saturation and blood signal contamination. Bland–Altman analyses were used to establish intersession measurement repeatability. A comparison of supine and prone positioning was made for two subjects, yielding similar results but superior comfort for lying supine. Mid‐septal myocardial PCr/ATP was 1.85 ± 0.37, with a repeatability coefficient of 17.7%. The repeatability coefficients for P_i_/ATP and PDE/ATP were 142.7% and 51.6%, respectively. Using the spatially homogeneous ^31^P spin excitation with the integrated whole‐body RF transmit coil, we made quantitative estimates of the myocardial energy status at a higher precision than previously achieved at lower main magnetic field strengths. The noninvasive nature and high precision of the presented ^31^P‐MR spectroscopic imaging approach will allow for therapeutic efficacy monitoring with repeated measurements in longitudinal study designs and investigations of normal physiology in healthy volunteers.

## Graphical abstract

We demonstrate the use of an integrated whole‐body phosphorus‐31 (^31^P) radiofrequency transmit coil for ^31^P‐MR spectroscopic imaging of the human heart at 7 T. Intersession measurement repeatability of the mid‐septal myocardial phosphocreatine (PCr) over adenosine triphosphate (ATP) concentration ratio in normal volunteers was 17.7%. The noninvasive nature and high precision will allow for therapeutic efficacy monitoring with repeated measurements and investigations of normal physiology in healthy volunteers.

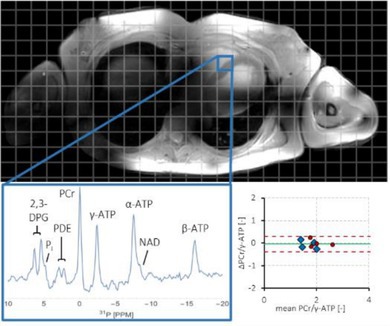

**List of abbreviations**
2,3‐DPG: 2,3‐diphosphoglycerate
^31^P‐MRS: phosphorus‐31 magnetic resonance spectroscopy
ATP: adenosine triphosphate
FWHM: full width at half maximum
GPC: glycerophosphocholine
GPE: glycerophosphoenthanolamine
ISIS: image‐selected in vivo spectroscopy
MR: magnetic resonance
NAD^+^/NADH: nicotinamide adenine dinucleotide
PCA: principal component analysis
PCr: phosphocreatine
PDE: phosphodiesters
P_i_
: inorganic phosphate
PSF: point spread function
RF: radiofrequency
SAR: specific absorption rate
SNR: signal‐to‐noise ratio
STEAM: stimulated echo acquisition mode
TE: echo time
TR: repetition time
UTE‐CSI: ultra‐short echo time chemical shift imaging

## 1 Introduction

Since the advent of magnetic resonance (MR) of the human body, the evaluation of in vivo myocardial high‐energy phosphate metabolism has been recognized as an important application of phosphorus‐31 MR spectroscopy (^31^P‐MRS) [1]. It allows the noninvasive detection and quantification of tissue phosphocreatine (PCr) and adenosine triphosphate (ATP) levels, which can be used to characterize the steady‐state in vivo myocardial energy status via the PCr over ATP concentration ratio. This parameter is sensitive to the impaired myocardial energy homeostasis that occurs in, for example, type 2 diabetes mellitus [2] and heart failure [3]. Although ^31^P‐MRS has been used over the past decades in various cross‐sectional [4] and longitudinal [5] studies of human myocardial (patho)physiology, this modality suffers from low sensitivity and poor measurement repeatability [6].

In contrast to well‐established MR methods to quantify myocardial function with MR imaging, ^31^P‐MRS of the heart remains a niche application in the versatile field of cardiovascular MR. Indeed, the intrinsically low sensitivity of ^31^P‐MRS and poor measurement precision hamper its broad application in the clinical workflow of disease diagnosis and therapeutic efficacy monitoring. The use of local ^31^P surface radiofrequency (RF) transmit/receive coils on conventional clinical MR systems limits the penetration depth of RF excitation pulses and subsequent signal acquisition that is needed to probe the deeper lying myocardial tissue. To mitigate these issues, some studies positioned their subjects in prone position over the surface coil, lowering the impact of respiratory motion while gravity brings the heart closer to the coil for improved sensitivity [1, 3, 4, 7, 8]. Nonetheless, non‐adiabatic inhomogeneous RF excitation complicates a quantitative evaluation of tissue metabolite content, while adiabatic RF excitation with a surface coil is hindered by a suboptimal signal‐to‐noise ratio (SNR) because of local specific absorption rate (SAR) restrictions [9]. Moreover, the use of surface RF transmit/receive coils may exacerbate contamination with signal from PCr and ATP in chest skeletal muscle because of the point spread function (PSF), which may further compromise the precision of ^31^P‐MRS‐based estimates of the myocardial energy status [6, 10].

Substantial effort has been put into improving sensitivity and signal localization for cardiac applications of ^31^P‐MRS, including the development of phased array receiver coils [11] and a whole‐body birdcage RF transmit coil insert [12]. Moreover, the theoretically proportional gain in SNR with increasing main magnetic field strength [13] has motivated a shift toward higher magnetic field strengths from 1.5 T [1] to 3 T [14] up to 7 T [15]. Indeed, SNR of PCr with ^31^P‐MRS at 7 T was 2.8‐fold higher than at 3 T in a paired comparison, while metabolite signal detection and quantification further benefited from the higher spectral resolution [15]. Recently, we reported on the use of an integrated whole‐body ^31^P RF transmit coil [16] in combination with a 16‐channel ^31^P receive array [17] for ^31^P‐MR spectroscopic imaging with full spatial coverage of the human liver at 7 T [18]. This setup combines homogeneous RF excitation over a large field of view with the sensitivity benefits of a high magnetic field strength. Although this configuration is suitable for ^31^P‐MRS throughout essentially the whole body, cardiac applications have not yet been demonstrated.

In this work, we report on the acquisition of ^31^P‐MR spectra of the in vivo human heart using a 7 T MR system equipped with an integrated whole‐body RF transmit coil for ^31^P spin excitation. We aimed to establish the intersession measurement repeatability of ^31^P‐MR spectroscopic imaging for quantifying the myocardial PCr/ATP ratio in normal volunteers, as an indicator of measurement precision of the myocardial energy status with ^31^P‐MRS.

## 2 Methods

This feasibility and repeatability study in normal volunteers was approved by the local medical research ethics committee (protocol number 15‐466; University Medical Center Utrecht, Utrecht, The Netherlands). All volunteers provided written informed consent before participation in this study. Eight subjects (male/female, *n* = 4/4) underwent the protocol as described below twice on the same day for an evaluation of intersession measurement repeatability. Additionally, two subjects (one male/one female) were examined both in supine and in prone position to assess the impact of supine versus prone positioning on data quality and signal quantification.

### 2.1 MR Protocol

Procedures were mostly similar to what has been described previously for ^31^P‐MR spectroscopic imaging of the human liver [18]. All MR examinations were performed with an upgraded whole‐body 7 T MR system (Philips, Best, The Netherlands) equipped with proton (^1^H) parallel transmission, integrated multi‐nuclei support, and an in‐house designed whole‐body double‐tuned phosphorus‐31/deuterium (^31^P/^2^H) quadrature birdcage RF transmit coil (inner diameter, 60 cm; transmit frequency for ^31^P, 120.6 MHz) embedded in the outside casing of the patient tube (Futura Composites, Heerhugowaard, The Netherlands) [16, 19]. This integrated transmit coil was powered by a two‐channel 2 × 15 kW RF amplifier (AN8137; Analogic Corporation, Peabody, MA, USA) to achieve a relatively homogeneous ^31^P B_1_^+^ transmit field of 10 μT throughout the body (spatial B_1_^+^ variation 19.2%) [19], allowing ^31^P‐MR spectroscopic imaging with whole‐body coverage. ^1^H RF transmission, signal reception, and ^31^P signal reception were done with an eight‐channel ^1^H transmit‐receive/16‐channel ^31^P receive array [17] consisting of eight elements, each with a pair of two geometrically decoupled ^31^P loop coils and one ^1^H dipole antenna [20]. These elements were positioned evenly around the subject's thorax to achieve essentially equal coverage for the anterior and posterior sides. Subjects were positioned supine and connected to a wireless peripheral pulse sensor unit (Invivo Corporation, Gainesville, FL, USA) with their left index finger.

After scout imaging, a five‐slice transversal stack of low‐flip angle gradient‐echo images was acquired for each ^1^H RF transmit channel to calculate the optimal RF phase setting through numerical calculations in MATLAB 2023b (The MathWorks Inc., Natick, MA, USA), maximizing the signal intensity averaged over a centered region (radius, 15 mm) within the volume of interest. These phase‐only RF shim settings were then used throughout the examination, with RF transmit amplitudes set equally for all channels. Next, image‐based B_0_ shimming [21] was performed on a 3D thoracic B_0_ map acquired at end‐expiration breath hold [18]. A nonlinear minimization algorithm was used to optimize first‐ and second‐order B_0_ shim settings for the main volume of interest encompassing the whole heart from apex to base. The rest of the thorax was also factored into the B_0_ shim setting calculations, although with an eightfold lower weight (MRCode; Tesla Dynamic Coils, Zaltbommel, The Netherlands) [21]. Then, a stack of cine series was acquired with an ECG‐triggered multi‐slice 2D gradient‐echo sequence in transversal orientation centered to the ^31^P‐MR spectroscopic imaging grid: field of view, 500 × 300 mm^2^; number of slices, 7; slice thickness/gap, 10/10 mm; repetition time (TR)/echo time (TE), 2.2/1.05 ms; flip angle, 15°; parallel imaging (compressed SENSE, sensitivity encoding) acceleration factor, 2; in‐plane resolution, 2 × 2 mm^2^; and number of reconstructed cardiac phases, 27. A transversal field of view rather than a more conventional oblique cardiac MR imaging plane (e.g., short axis) was chosen to allow for a smaller matrix for 3D ^31^P‐MR spectroscopic imaging, reducing acquisition time while still avoiding fold‐in artefacts.

Finally, ^31^P‐MR spectra were acquired with a 3D phase‐encoded ^31^P‐MR spectroscopic imaging sequence using Hamming‐weighted *k*‐space sampling: field of view, 500 (left–right) × 300 (anterior–posterior) × 340 (feet–head) mm^3^; matrix, 25 × 15 × 17; isotropic resolution, 20 × 20 × 20 mm^3^; TR, 60 ms; block pulse RF excitation (10 μT; 0.1984 ms; 95% excitation bandwidth, 1823 Hz; PCr at 0.00 ppm on‐resonance) for a 12° flip angle; acquisition delay, 0.50 ms; spectral bandwidth, 5000 Hz; number of points, 256; number of signal averages, 20 in the center of the Hamming‐weighted *k*‐space sampling scheme; acquisition time, 20 min 59 s; and free breathing with no respiratory gating. A noise scan was acquired (number of points, 8192; number of signal averages, 10) with the power for the ^31^P RF pulses turned off.

### 2.2 MR Data Analyses

All ^31^P‐MR data were processed offline in MATLAB 2023b (The MathWorks Inc.) according to the procedures outlined previously [18]. The weighing coefficients for *k*‐space locations were corrected with an additional correction factor to match the discrete number of acquisitions in the Hamming‐weighted sampling scheme with the ideal Hamming window. Signals were then Fourier‐transformed in the spatial domain, followed by zero‐ and first‐order phase corrections. A set of virtual channels with decorrelated (i.e., whitened) noise was generated from the signals of each ^31^P receive channel and the noise scan [22]. Subsequently, principal component analysis (PCA)‐based denoising [23] was applied to the spatiotemporal data using patches of 5 × 5 × 5 voxels (Figure S1). The truncation threshold was determined automatically using the Marchenko–Pastur distribution. After denoising, the Roemer equal‐noise algorithm [24] was used for channel combination. The resultant coil sensitivities were also used for channel combination of the non‐PCA‐denoised data to allow for an evaluation of the effect of denoising in a direct comparison between denoised and non‐denoised data following identical processing procedures. Apodization (20 Hz) and zero‐filling (to 512 points) were applied for visualization of spectra only.

Transversal cine MR images of the thorax were used to define myocardial voxels within the 3D ^31^P‐MR spectroscopic imaging grid, by selecting those voxels that co‐localized with the myocardium (i.e., septum and posterior wall) in two mid‐ventricular slices at end‐diastole. To minimize PSF‐induced contamination with signal from the liver, these slices were defined as either two slices above (i.e., more cranial) the most cranial slice containing tissue from the left liver lobe in the ^1^H‐MR image or one slice above the most caudal slice containing predominantly heart tissue (Figure S2). For single‐voxel analyses, we selected the slice positioned at least one slice above the most caudal slice containing predominantly heart tissue.

SNR was calculated as the height of the real part of the α‐ATP signal peak in the frequency domain after appropriate phasing divided by the standard deviation (SD) of the noise (i.e., 10–20 ppm range) in the frequency domain.

Signal quantification was based on a fitting model that included biochemical, physical, and empirical prior knowledge. Fitting was done per voxel in the time domain using AMARES in MATLAB 2023b [25] integrated in the open‐source MR spectroscopy analysis toolbox OXSA [26]. Lorentzian line shapes were fitted to nine resonance peaks: a triplet to the β‐ATP group at −16.04 ppm, nicotinamide adenine dinucleotide (NAD^+^/NADH) at −8.21 ppm, a doublet to α‐ATP at −7.52 ppm, a doublet to γ‐ATP at −2.43 ppm, phosphocreatine (PCr) at 0.00 ppm, phosphodiesters (PDE) glycerophosphocholine (GPC) and glycerophosphoenthanolamine (GPE) at 2.17 and 2.94 ppm, respectively, inorganic phosphate (P_i_) at 4.92 ppm, and a doublet [27] to the 2,3‐diphosphoglycerate (2,3‐DPG) signal from the blood at 5.82 ppm. The *J*‐coupling constant for the ATP signals was fixed at 16 Hz, and their line widths were kept equal. Line widths for both PDE resonances were kept equal. The splitting constant for the 2,3‐DPG doublet peaks was set at 113 Hz, with their line width and amplitude kept equal. Chemical shifts were constrained by a 0.6 ppm margin around their initial values for each resonance peak, while their line widths were constrained between 10 and 100 Hz. The first‐order phase was fixed (based on the acquisition delay), and only the zero‐order phase (one value for all signals) was allowed to vary.

Magnetic field B_0_ homogeneity was assessed through the line width (full width at half maximum, FWHM) of the PCr signal. Corrections for partial saturation were based on the known TR of 60 ms and 12° flip angle and literature values for longitudinal relaxation T_1_ time constants of human myocardium at 7 T: T_1_ for PCr, 3.09 s; γ‐ATP, 1.82 s; α‐ATP, 1.39 s; β‐ATP, 1.02 s; 2,3‐DPG, 3.05 s [15]; and P_i_, 5 s [28]. Because the T_1_ relaxation time constant of myocardial PDE is unknown, the PDE/γ‐ATP ratio was not corrected for partial saturation. The contribution of signals from ATP, PDE, and P_i_ in the blood was estimated relative to the measured 2,3‐DPG signal based on ratios published previously for healthy volunteers [29]: β‐ATP/2,3‐DPG, 11%; PDE/2,3‐DPG, 18%; and P_i_/2,3‐DPG, 5%. Myocardial metabolite content, corrected for partial saturation and blood signal contamination, was then expressed relative to the γ‐ATP signal amplitude, with the PCr/γ‐ATP ratio serving as a measure of the myocardial energy status. One value per subject per session was calculated by averaging the quantification results for the myocardial voxels defined on the anatomical reference images as a “global” parameter estimate for the myocardium. Single‐voxel analyses were conducted for a mid‐septal voxel. With the fixed B_1_^+^ transmit field that was calibrated previously [19], small deviations in effective flip angle may occur within the myocardium. Analysis shows that flip angle deviations of ± 25% from 12° would result in only a modest over‐ or underestimation (+8% to −9%) for the myocardial PCr/γ‐ATP ratio corrected for partial saturation and blood signal contamination (Figure S3).

### 2.3 Statistical Analyses

Data are reported as mean ± between‐subject SD for the first session. Intersession measurement repeatability was assessed with Bland–Altman analyses [30], with the repeatability coefficient defined as 1.96 times the SD of the signed difference (*Δ*) between two repeated measurements in each subject and expressed as a percentage of the group mean value (*μ*): repeatability coefficient (%) = [SD(*Δ*) × 1.96] /*μ* × 100%, with *μ* the overall group mean and *Δ* the signed difference between two repeated measurements (*m*) per subject: *Δ* = *m*_1_ − *m*_2_.

## 3 Results

Eight volunteers (male/female, *n* = 4/4; age, 31 ± 9 years; body mass index, 22.6 ± 2.9 kg/m^2^) were scanned twice. After the first session, the subjects were removed from the scanner for a 15‐min break and repositioned for the second session. A single session took approximately 45 min to complete and was tolerated well by all volunteers.

**FIGURE 1:**
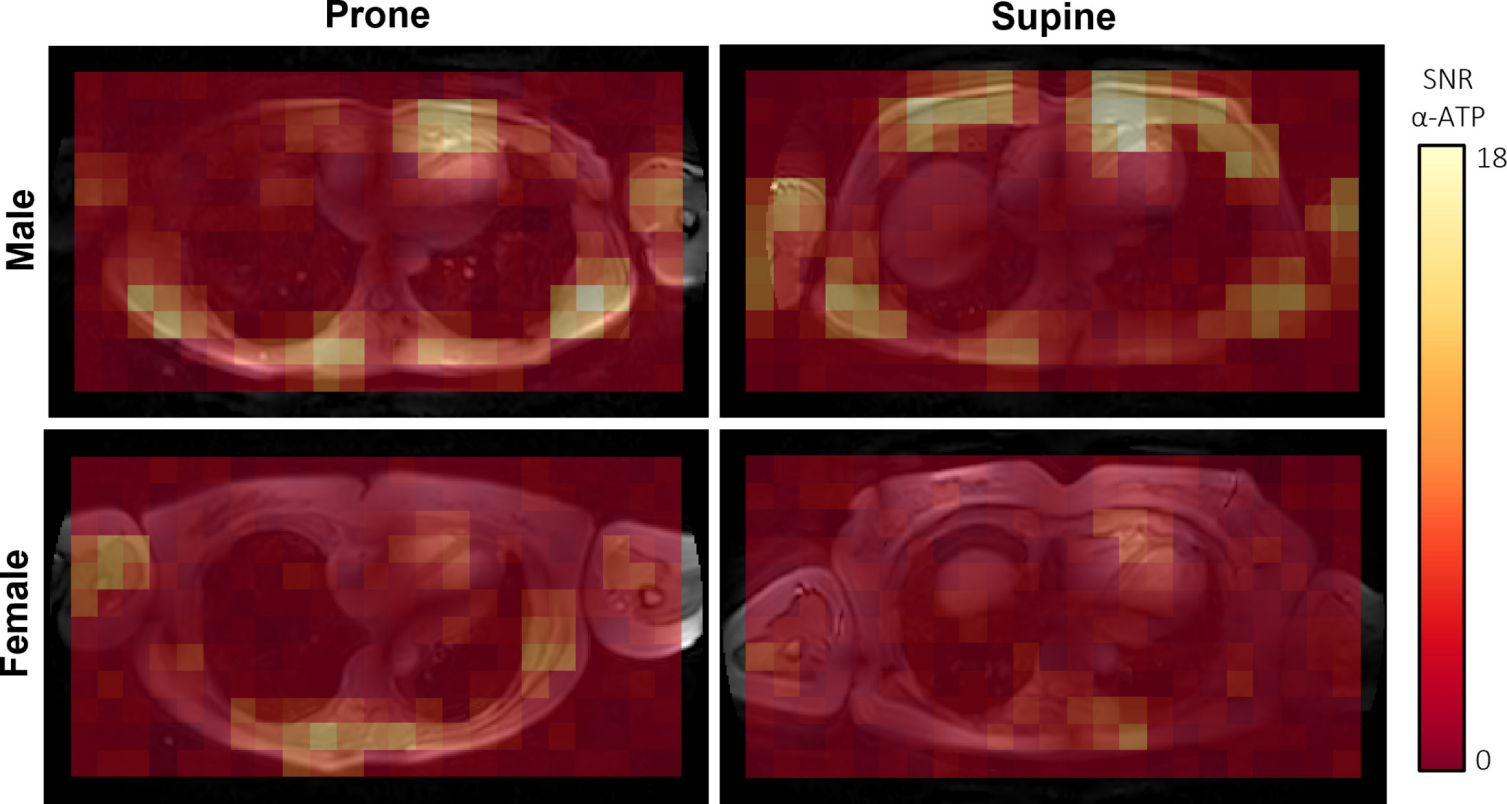
Signal‐to‐noise ratio (SNR) for α‐adenosine triphosphate (α‐ATP) in data without prior denoising mapped onto a transversal mid‐ventricular cine MR image of the thorax in a male (top row) and a female (bottom row) subject, in prone (left column) and in supine (right column) position.

First, we pragmatically compared the practicality and sensitivity of our ^31^P‐MRS approach for two subjects (male/female, *n* = 1/1) lying either in prone or in supine position. SNR for α‐ATP was similar in both positions (Figure 1), while subject comfort was considerably worse in the prone position. Given the adequate SNR achieved in the supine position, and the superior comfort of lying supine rather than prone, we conducted the examinations for our evaluation of measurement repeatability with subjects positioned supine.

**FIGURE 2:**
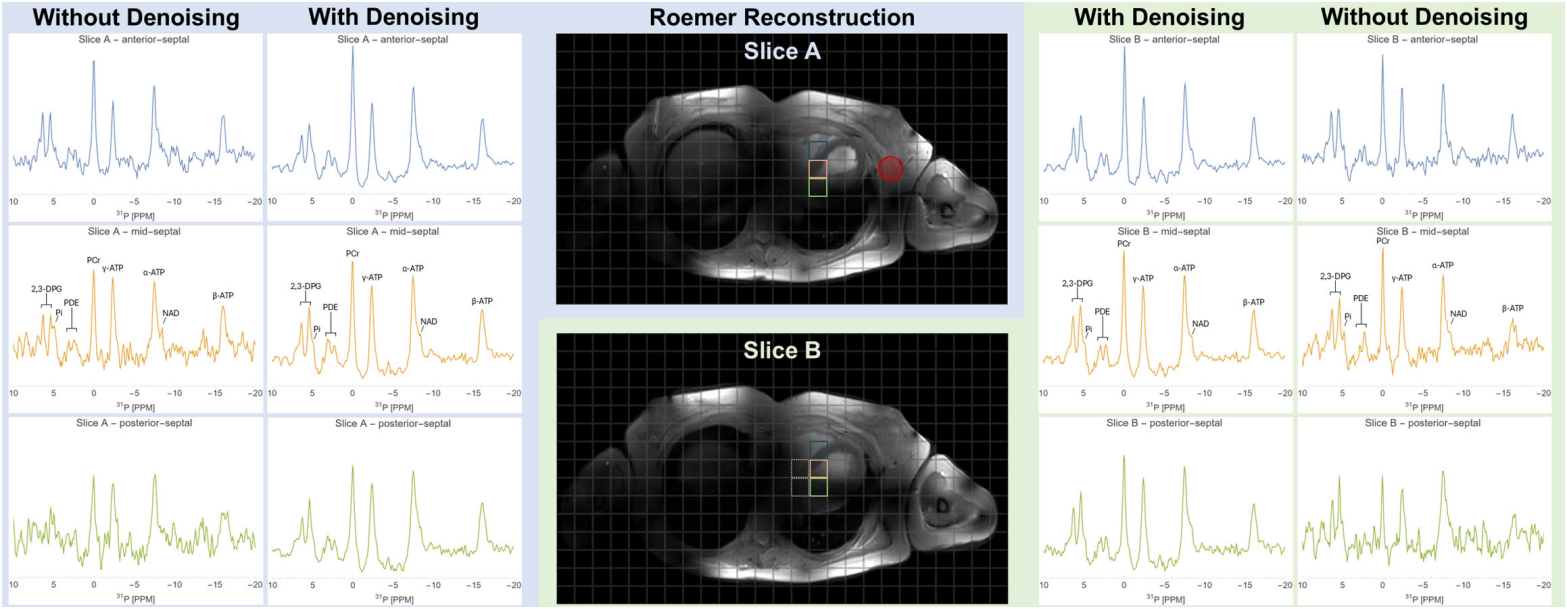
Phosphorus‐31 magnetic resonance (^31^P‐MR) spectra of 20 × 20 × 20 mm^3^ myocardial voxels obtained at 7 T in a normal volunteer (female; age, 52 years; body mass index, 24.6 kg/m^2^) in supine position. The 3D ^31^P‐MR spectroscopic imaging grid is overlaid on ECG R‐wave triggered cine MR images of the thorax for two mid‐ventricular transversal slices (A, B) at end‐diastole. Spectra within each column are scaled to equalize the power of the noise. Innermost columns show spectra after Roemer channel combination *with* subsequent principal component analysis (PCA)‐based denoising, while the outermost columns show spectra *without* denoising. Note the resonance peak for phosphocreatine (PCr) at 0.00 ppm, the three multiplets (α‐, β‐, γ‐) for adenosine triphosphate (ATP) with an upfield shoulder near α‐ATP arising from nicotinamide adenine dinucleotide (NAD) signal, two resonances for phosphodiesters (PDE), and 2,3‐diphosphoglycerate (2,3‐DPG) signal from ventricular blood shouldered by signal from inorganic phosphate (P_i_). The resonance peak at −5 ppm is not assigned [1]. Dotted lines indicate voxels that were also included for estimating the myocardial PCr/γ‐ATP for this particular subject (corresponding ^31^P‐MR spectra not visualized here). The red circle outlines the effective voxel size at 64% of the point spread function (PSF) area [31].

Figure 2 shows ^31^P‐MR spectra for single voxels across the myocardium of a single male subject after PCA‐based denoising as well as without prior denoising, revealing a prominent resonance peak for PCr, the three multiplets for ATP with an upfield shoulder near α‐ATP arising from NAD^+^/NADH signal, two resonances for PDEs, and 2,3‐DPG signal from ventricular blood shouldered by signal from P_i_. In the non‐denoised data, SNR appeared to gradually decrease from the anterior voxels to the more posterior voxels, while denoising effectively reduced the noise in all voxels. Notably, the more anterior voxels featured a more prominent PCr signal, which can likely be attributed to some contamination with signal from PCr in chest skeletal muscle.

**FIGURE 3:**
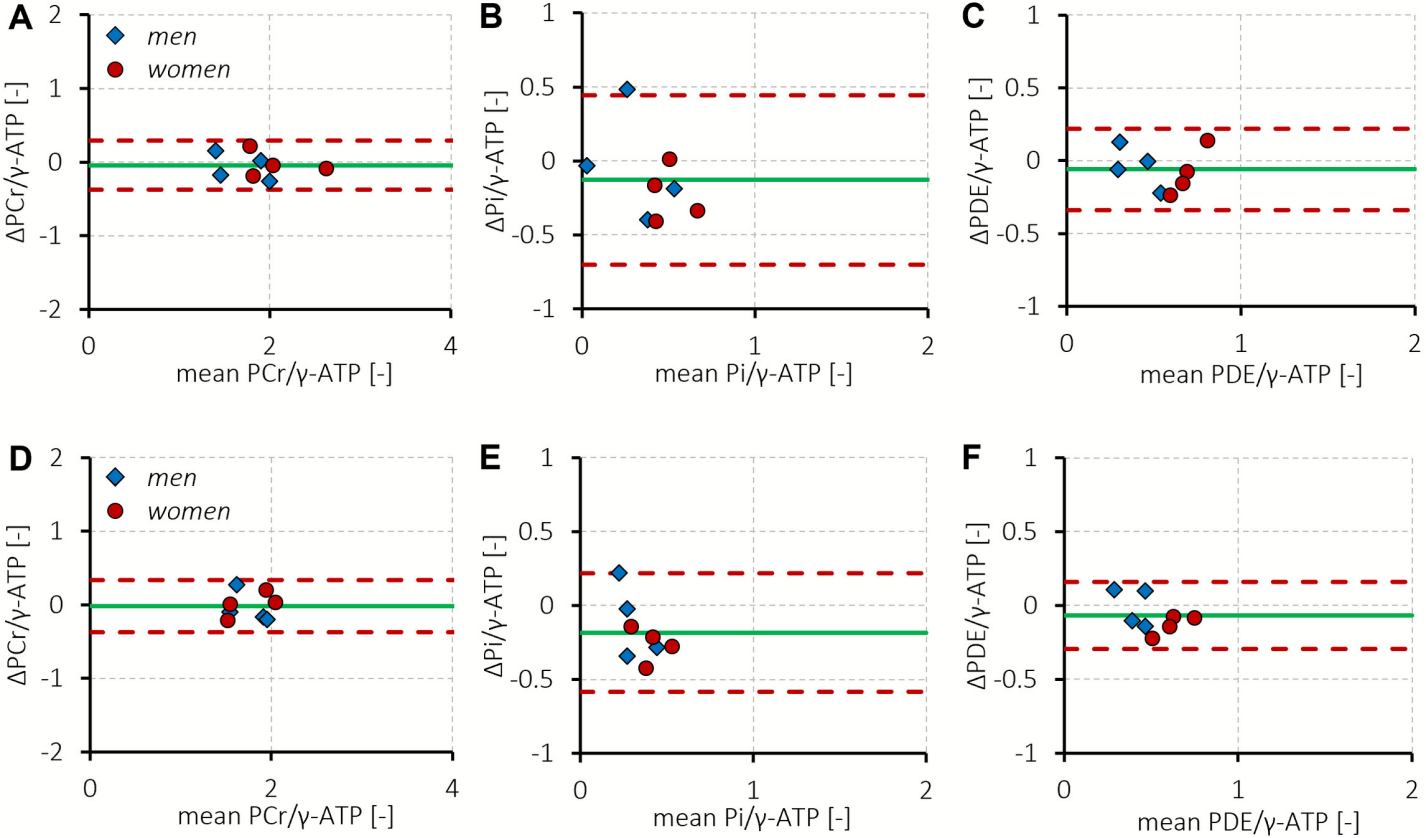
Bland–Altman analysis of the intersession measurement repeatability for single‐voxel septal (A–C) and mean myocardial (D–F) PCr/γ‐ATP (A, D), P_i_/γ‐ATP (B, E), and PDE/γ‐ATP (C, F) measured with ^31^P‐MR spectroscopic imaging at 7 T in men (*n* = 4; blue diamonds) and women (*n* = 4; red circles). Results are based on spectral fits with prior principal component analysis (PCA)‐based denoising. The dashed red lines represent the 95% confidence interval at 1.96 times the standard deviation of the mean difference (green solid line) between two consecutive measurements on the same day. The intersession repeatability coefficient for septal PCr/γ‐ATP (A) was 17.7% at an overall mean value of 1.88 ± 0.38 with a mean difference between two measurements of −0.04 ± 0.17. The intersession repeatability for septal P_i_/γ‐ATP (B) was 142.7% at an overall mean value of 0.40 ± 0.19 with a mean difference of −0.13 ± 0.29. For septal PDE/γ‐ATP (C), the intersession repeatability coefficient was 51.6% at an overall mean value of 0.54 ± 0.18 and a mean difference of −0.06 ± 0.14. Septal PDE/γ‐ATP was higher in women than in men (*p* = 0.030). The intersession repeatability coefficient for mean myocardial PCr/γ‐ATP (D) was 20.3% at an overall mean value of 1.76 ± 0.22 with a mean difference between two measurements of −0.02 ± 0.18. The intersession repeatability for mean myocardial P_i_/γ‐ATP (E) was 113.9% at an overall mean value of 0.35 ± 0.11 with a mean difference of −0.18 ± 0.20. For PDE/γ‐ATP (F), the intersession repeatability coefficient was 44.4% at an overall mean value of 0.51 ± 0.14 and a mean difference of −0.07 ± 0.12.

The mean SNR of α‐ATP for our subjects (*n* = 8) was 5.5 ± 1.0 without prior denoising. Mean FWHM line width for PCr was 26.0 ± 4.7 Hz, indicating that good B_0_ shim quality was achieved. The myocardial energy status in the septum, expressed as PCr/γ‐ATP after correction for partial saturation and blood signal contamination, and based on spectral fits after PCA‐based denoising, was 1.85 ± 0.37. The intersession repeatability coefficient for single‐voxel septal PCr/γ‐ATP was 17.7% (Figure 3A). Myocardial inorganic phosphate content in the septum, estimated via P_i_/γ‐ATP, was 0.34 ± 0.18, with a repeatability coefficient of 142.7% (Figure 3B). Myocardial PDE/γ‐ATP in the septum was 0.51 ± 0.19, which was higher in women (0.65 ± 0.17) than in men (0.38 ± 0.09; two‐sided Student's *t*‐test, *p* = 0.030), and was measured with a repeatability coefficient of 51.6% (Figure 3C).

Per subject, six to nine voxels were selected that co‐localized with the myocardium, yielding a mean nominal tissue volume of 60 ± 7 mL for mean myocardial signal quantification. The mean (i.e., global) myocardial energy status, expressed as PCr/γ‐ATP after correction for partial saturation and blood signal contamination averaged over multiple myocardial voxels, was 1.75 ± 0.25. The intersession repeatability coefficient for mean myocardial PCr/γ‐ATP was 20.3% (Figure 3D). Mean myocardial P_i_/γ‐ATP was 0.26 ± 0.10, with a repeatability coefficient of 113.9% (Figure 3E). Mean myocardial PDE/γ‐ATP was 0.48 ± 0.13 and was measured with a repeatability coefficient of 44.4% (Figure 3F).

Measurement precision for metabolite ratios based on non‐denoised data was substantially worse (i.e., higher intersession repeatability coefficients), with a repeatability coefficient of 41.0% for single‐voxel septal PCr/γ‐ATP and 47.8% for mean myocardial PCr/γ‐ATP (Figure S4). As such, denoising was beneficial for precision in terms of measurement repeatability. In general, measurement repeatability was highest for the prominent PCr signal, while quantification of myocardial P_i_ content could only be done with low precision.

## 4 Discussion

Quantification of the in vivo myocardial energy status can be an important readout for investigations of cardiovascular physiology, heart failure pathogenesis, and treatment efficacy [32]. The present work demonstrated how estimates of the myocardial PCr/ATP concentration ratio can be obtained noninvasively using ^31^P‐MRS at 7 T. With an integrated whole‐body ^31^P RF transmit coil and 16‐channel ^31^P receive array, we achieved a precision in terms of intersession measurement repeatability that would require a change of > 20% in myocardial PCr/γ‐ATP within a single subject to attribute its detection with 95% confidence to (patho)physiological effects rather than measurement error. As such, measurement precision is not sensitive enough to detect subtle changes on an individual‐patient basis, which would be needed for any meaningful diagnostic application of ^31^P‐MRS of the heart. Instead, a power calculation shows that for detecting a 0.2‐point decrease in the myocardial PCr/γ‐ATP ratio with a power of 80% [8, 33], a relatively small cohort of only *n* = 9 subjects would be needed with the measurement repeatability presented here.

Measurement repeatability of human myocardial PCr/ATP has been reported previously for several different main magnetic field strengths. Initial work at 1.5 T [34] and 2 T [35] achieved repeatability coefficients of > 45% (voxel size, 49 mL) and 22% (voxel size not reported), respectively. At 3 T, repeatability coefficients exceeded 40% [6] up to 53% [8]. Using their 28‐min 3D ultra‐short TE chemical shift imaging (UTE‐CSI) protocol with 15 × 15 × 25 mm^3^ voxels [15], Ellis et al. achieved a repeatability coefficient of 26% for measuring myocardial PCr/ATP in a septal voxel at 7 T [33]. By increasing voxel size and decreasing the number of averages, this protocol was shortened to 6.5 min, at the expense of a lower measurement precision that was similar to 3‐T results (repeatability coefficient, 44%) [33]. Further shortening of the 3D ^31^P‐MR spectroscopic imaging acquisition time using concentric ring trajectories for accelerated *k*‐space sampling down to 2.5 min and even 1.5 min led to a further reduction in intersession measurement precision for myocardial PCr/ATP (i.e., higher repeatability coefficients, > 91%) [36]. Those prior reports used surface ^31^P RF coils, either for both RF transmission and signal reception by a single coil [6, 34, 35] or separately by a single large RF transmit coil and multiple receive coils [8, 33, 36]. Along this line, a dipole‐loop system was recently developed that comprised four ^31^P dipole antennas for RF transmission and 16 loops for signal reception [37]. That setup allowed for spatially resolved ^31^P‐MRS of the left ventricular myocardial wall with intersession repeatability coefficients ranging from 21% in the inferior wall up to 42% in the lateral wall [37]. While our protocol involved slightly larger voxels than the smallest voxels defined in prior work at 7 T [15, 33] (8 vs. 5.6 mL), the use of a whole‐body birdcage ^31^P RF transmit coil in combination with a 16‐channel ^31^P receive array and PCA‐based denoising enabled us to achieve a PCr/ATP repeatability coefficient of < 18% at an acquisition time of < 21 min.

Skeletal muscle contains substantially higher levels of PCr compared to the myocardium, and signal from PCr in chest skeletal muscle may therefore contaminate spectra acquired from the heart. Such signal contamination may be particularly relevant in studies that use surface coils with inhomogeneous RF excitation. To mitigate this issue, any ^31^P signal from metabolites in chest skeletal muscle can be saturated with saturation bands [8, 15, 37], effectively suppressing signal from outside the volume of interest. Alternatively, the use of adiabatic pulses can help reduce the spatial variation of RF excitation to effect consistent PCr/ATP values across the myocardial wall [10]. Saturation pulses and adiabatic pulses are highly SAR‐demanding, and their use may not be favorable at 7 T. Here, we did not use any saturation bands or adiabatic pulses. Yet, the present estimated normal mean myocardial PCr/ATP ratios corrected for partial saturation and blood signal contamination (1.75 ± 0.25) are in keeping with literature mean values for normal myocardial PCr/ATP in humans [38], as well as with prior work at 7 T that used suppression of chest skeletal muscle signal, if not somewhat lower (i.e., 1.9 [33] to 2.1 [8, 10, 15]).

Earlier work, predominantly performed at 1.5 T and 3 T, involved large voxels that enclosed the entire left ventricle using single‐voxel 3D image‐selected in vivo spectroscopy (ISIS) [6, 34] or relatively large voxels with 1D spectroscopic imaging [4, 35]. The 3D ^31^P‐MR spectroscopic imaging sequences applied here and elsewhere [33, 37] may allow for a spatially resolved evaluation of the myocardial energy status. This will be relevant in investigations of conditions where myocardial energy homeostasis may be affected differentially across myocardial segments, for example, by perfusion deficits. Future work in such patient cohorts is needed to establish the sensitivity and added value of high spatial resolution 3D ^31^P‐MRS. Given that ^31^P‐MRS is essentially the only method to quantify the in vivo myocardial energy status in humans, a validation of detecting any regional differences against biochemical assays of high‐energy phosphate metabolite concentrations in myocardial tissue will be very difficult, if not impossible. We previously compared single‐voxel 3D ISIS‐based ^31^P‐MRS of the in vivo mouse heart against spectrophotometric assays of PCr and ATP concentrations in ex vivo samples [39], validating that a ^31^P‐MRS‐based estimate of the global myocardial energy status is sensitive to perturbations in myocardial energy homeostasis. As such, the PCr/ATP ratio as a global indicator of the myocardial energy status, measured precisely and likely also accurately, will contribute to further our understanding of human myocardial metabolism and (patho)physiology [32].

We observed a notably higher myocardial PDE content in women compared to men in this small cohort of normal volunteers. The relevance of PDE in myocardial (patho)physiology is essentially unknown. Elevated myocardial PDE was observed in patients with dilated cardiomyopathy and could be related to elevated turnover rates of cardiomyocyte membranes [40]. Alternatively, based on measurements in hearts of freshwater turtles, it has been postulated that cytosolic PDE may play a beneficial role in myocardial hypoxia tolerance [41]. With a reasonable measurement precision for quantifying myocardial PDE/γ‐ATP, ^31^P‐MRS at 7 T can now be a noninvasive tool to investigate myocardial PDE in the in vivo human heart.

### 4.1 Limitations

We note some limitations of our method as presented here. While precisely estimating myocardial PCr/ATP on a whole‐heart level as well as for the mid‐ventricular septum, the current approach is not expected to be sensitive to regional differences in myocardial energy metabolism. Our 20‐mm nominal isotropic voxels are relatively large, given that the healthy LV myocardial wall thickness is less than 12 mm [42]. Moreover, we did not account for any respiratory or cardiac motion in our 3D ^31^P‐MR spectroscopic imaging acquisition or data processing. Combined, this will inevitably lead to partial voluming and signal bleeding via a coarse PSF. To mitigate signal contamination from (moving) chest skeletal muscle, we excluded anterior voxels from our quantifications. Note that while cardiac triggering can increase ^31^P‐MRS signal amplitudes and improve fit quality [43], it may not improve measurement repeatability [44]. In addition, violating the steady‐state of magnetization condition with a variable TR due to cardiac triggering or respiratory gating may complicate corrections for partial saturation. To mitigate the limited B_1_^+^ efficiency of the whole‐body ^31^P RF transmit coil while ensuring a broad excitation bandwidth and optimal SNR per unit time, we used a low‐flip angle (12°), short‐TR (60 ms) 3D ^31^P MR spectroscopic imaging sequence. Employing the longer (and inevitably variable) TR that would come with cardiac triggering (ECG R‐R interval of approximately 1 s in humans) necessitates the use of higher flip angles (e.g., Ernst angle) that would require longer RF pulses with narrower bandwidths. As such, we expect that introducing cardiac triggering into the current sequence would lengthen acquisition time without offering a substantial gain in measurement precision. Novel acceleration techniques combined with cine‐like signal binning [45] may be a promising approach to further improve the performance of 3D ^31^P‐MR spectroscopic imaging for cardiac applications.

We could not unambiguously detect myocardial P_i_ signal and estimated myocardial P_i_/ATP with low precision. Partial voluming and signal bleeding contributed to the remaining 2,3‐DPG signal arising from ventricular blood in myocardial spectra. Despite the increased spectral resolution at a 7 T magnetic field strength, overlap with 2,3‐DPG resonances prevents a clear detection of myocardial P_i_. Promising approaches for detecting and quantifying P_i_ include a long‐TR ^31^P‐MR spectroscopic imaging sequence that prevents saturation of the P_i_ signal relative to flowing ventricular blood, yielding a P_i_/PCr measurement repeatability coefficient of 33% [28]. Alternatively, a single‐voxel stimulated echo acquisition mode (STEAM) sequence for localized ^31^P‐MR signal acquisition from the septum has been proposed (P_i_/PCr repeatability coefficient, 54%), using a mixing time that effectively suppresses signal from 2,3‐DPG in flowing blood [46]. Note that STEAM localization requires 90° RF pulses, which are SAR‐demanding and may not be achievable with our whole‐body ^31^P RF transmit coil.

In conclusion, we established a protocol for the noninvasive quantification of the myocardial energy status in the in vivo human heart using an integrated whole‐body ^31^P RF transmit coil for ^31^P‐MRS at 7 T. Precision, assessed by intersession measurement repeatability of the myocardial PCr/ATP concentration ratio, has improved over prior work at 3 T. As such, this approach may be suitable for quantitative evaluations of human myocardial energy metabolism in health and disease. Its noninvasive nature will readily allow for therapeutic efficacy monitoring with repeated measurements in longitudinal study designs or investigations of normal physiology in healthy volunteers.

## Author Contributions

Mark W.J.M. Gosselink and Jeanine J. Prompers designed the study. Dennis W.J. Klomp and Adrianus J. Bakermans obtained funding for this study. Jeanine J. Prompers supervised all the work. Mark W.J.M. Gosselink and Kathy Verkerk performed the experiments. Mark W.J.M. Gosselink, Martijn Froeling, Kathy Verkerk, Adrianus J. Bakermans, and Jeanine J. Prompers analyzed the results. Mark W.J.M. Gosselink, Adrianus J. Bakermans, and Jeanine J. Prompers produced the figures. Adrianus J. Bakermans wrote the first draft of the manuscript. All authors edited the manuscript and approved the final version.

## Funding

This work was supported by the United States National Institutes of Health (R01 HL173346) and HORIZON EUROPE European Innovation Council (101058229).

## Supporting information


**Figure S1:** Simulation of denoising performance. Principal‐component analysis (PCA)‐based denoising was performed on an in silico 3D phantom, encompassing three distinct compartments (A) representing metabolite content for (B) background (blue, no signal), skeletal muscle (green, high level of phosphocreatine (PCr)), and liver (red, no PCr). Spectra are shown for three adjacent voxels (C). Spectra (black) were simulated at 7 T (bandwidth, 5 kHz; echo time, 0 ms; number of points, 512; line width, 50 Hz) without noise, with a signal‐to‐noise ratio (SNR) for muscle PCr of 200, and an SNR for muscle PCr of 20 (D), while noise power was kept equal between compartments for each set. PCA‐based denoising was then applied with a 5 × 5 × 5 kernel identical to the processing of our in vivo data. Comparison of original (black) and denoised (red) spectra demonstrates signal recovery (D). Note how noise is effectively reduced in all compartments, while there is essentially no visible mixing or blurring of the high PCr signal from skeletal muscle into the background or liver voxels post‐denoising, indicating that spatial signal bleeding between tissue compartments due to PCA‐based denoising is negligible.
**Figure S2:** Effect of contamination with signal arising from the liver in voxels adjacent to the heart. Note how only little signal contamination from phosphocreatine (PCr, 0.00 ppm) from the heart or muscle appears in a voxel (yellow) selected within the liver in the more caudal slice (F) and that the PCr signal increases in contingent slices in the cranial direction (H). In the two most cranial slices (outlined in green), no liver is visible in the subject's left side in the proton MR image, and the ^31^P‐MR spectra are characteristic for myocardial tissue. To minimize any contamination with signal from the liver, heart slices for defining myocardial ^31^P‐MR spectroscopic imaging voxels were selected conservatively: either two slices above (i.e., more cranial) the most cranial slice containing tissue from the left liver lobe in the proton MR image or one slice above the most caudal slice containing predominantly heart tissue. In this example, the mid‐septal voxel for single‐voxel analyses was selected in the most cranial slice. Nominal voxel size for ^31^P‐MR spectroscopic imaging, 20 × 20 × 20 mm3; proton MR image slice thickness/gap, 10/10 mm.
**Figure S3:** Effect of B_1_^+^ (i.e., effective flip angle) variation on the phosphocreatine (PCr) to γ‐adenosine triphosphate (γ‐ATP) ratio, only corrected for partial saturation (blue dashed line) or corrected for both partial saturation as well as for blood signal contamination (red line). The latter was determined for the full range of voxel blood contributions (based on the 2,3‐DPG signal per voxel) as encountered in our study (indicated by the black error bars). A ± 25% variation in the effective flip angle will lead to mean deviations of only −9% to +8% in PCr/γ‐ATP corrected for partial saturation and blood signal contamination.
**Figure S4:** Bland–Altman analysis of the intersession measurement repeatability for single‐voxel septal (A–C) and mean myocardial (D–F) PCr/γ‐ATP (A, D), Pi/γ‐ATP (B, E), and PDE/γ‐ATP (C, F) measured with ^31^P‐MR spectroscopic imaging at 7 T in men (n = 4; blue diamonds) and women (n = 4; red circles). Results are based on spectral fits without prior denoising. The dashed red lines represent the 95% confidence interval at 1.96 times the standard deviation of the mean difference (green solid line) between two consecutive measurements on the same day. The intersession repeatability coefficient for septal PCr/γ‐ATP (A) was 41.0% at an overall mean value of 1.65 ± 0.33 with a mean difference between two measurements of −0.08 ± 0.35. The intersession repeatability for septal Pi/γATP (B) was 202.3% at an overall mean value of 0.30 ± 0.27 with a mean difference of −0.06 ± 0.31. For septal PDE/γATP (C), the intersession repeatability coefficient was 111.3% at an overall mean value of 0.38 ± 0.25 and a mean difference of −0.03 ± 0.22. The intersession repeatability coefficient for mean myocardial PCr/γ‐ATP (D) was 47.8% at an overall mean value of 1.61 ± 0.22 with a mean difference between two measurements of 0.00 ± 0.39. The intersession repeatability for mean myocardial Pi/γ‐ATP (E) was 122.3% at an overall mean value of 0.33 ± 0.11 with a mean difference of −0.08 ± 0.20. For PDE/γ‐ATP (F), the intersession repeatability coefficient was 81.4% at an overall mean value of 0.38 ± 0.18 and a mean difference of −0.06 ± 0.16.

## Data Availability

The data that support the findings of this study are available from the corresponding author upon reasonable request.
