# Supplementary material for: 31P‐MRS of the Human Heart at 7 T With an Integrated Whole‐Body 31P Radiofrequency Transmit Coil
Source: NMR Biomed. 2026 Feb 19;39(4):e70248. doi: 10.1002/nbm.70248 (PMC12921427; doi:10.1002/nbm.70248)
Supplement: Supplementary file 1 — Figure S1: Simulation of denoising performance. Principal‐component analysis (PCA)‐based denoising was performed on an in silico 3D phantom, encompassing three distinct compartments (A) representing metabolite content for (B) background (blue, no signal), skeletal muscle (green, high level of phosphocreatine (PCr)), and liver (red, no PCr). Spectra are shown for three adjacent voxels (C). Spectra (black) were simulated at 7 T (bandwidth, 5 kHz; echo time, 0 ms; number of points, 512; line width, 50 Hz) without noise, with a signal‐to‐noise ratio (SNR) for muscle PCr of 200, and an SNR for muscle PCr of 20 (D), while noise power was kept equal between compartments for each set. PCA‐based denoising was then applied with a 5 × 5 × 5 kernel identical to the processing of our in vivo data. Comparison of original (black) and denoised (red) spectra demonstrates signal recovery (D). Note how noise is effectively reduced in all compartments, while there is essentially no visible mixing or blurring of the high PCr signal from skeletal muscle into the background or liver voxels post‐denoising, indicating that spatial signal bleeding between tissue compartments due to PCA‐based denoising is negligible. Figure S2: Effect of contamination with signal arising from the liver in voxels adjacent to the heart. Note how only little signal contamination from phosphocreatine (PCr, 0.00 ppm) from the heart or muscle appears in a voxel (yellow) selected within the liver in the more caudal slice (F) and that the PCr signal increases in contingent slices in the cranial direction (H). In the two most cranial slices (outlined in green), no liver is visible in the subject's left side in the proton MR image, and the 31P‐MR spectra are characteristic for myocardial tissue. To minimize any contamination with signal from the liver, heart slices for defining myocardial 31P‐MR spectroscopic imaging voxels were selected conservatively: either two slices above (i.e., more cranial) the most cranial slic [file NBM-39-e70248-s001.pdf]

Supplementary Material

^{31}P-MRS of the human heart at 7 T with an integrated whole-body ^{31}P radiofrequency transmit coil

Mark W.J.M. Gosselink, Martijn Froeling, Kathy Verkerk, Dennis W.J. Klomp, Adrianus J. Bakermans, Jeanine J. Prompers.

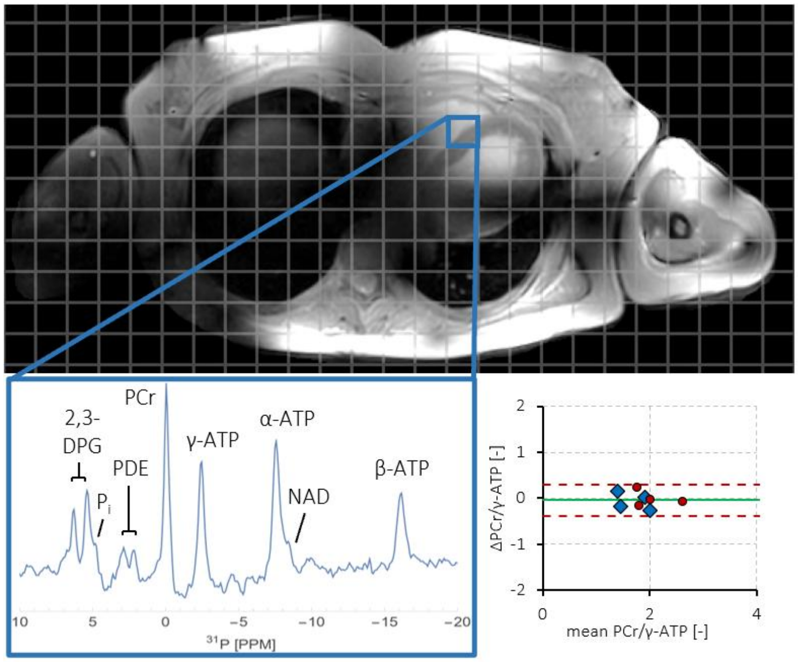

Graphical Abstract. We demonstrate the use of an integrated whole-body phosphorus-31 (^{31}P) radiofrequency transmit coil for ^{31}P-MR spectroscopic imaging of the human heart at 7 T. Inter-session measurement repeatability of the mid-septal myocardial phosphocreatine (PCr) over adenosine triphosphate (ATP) concentration ratio in normal volunteers was 17.7%. The noninvasive nature and high precision will allow for therapeutic efficacy monitoring with repeated measurements, and investigations of normal physiology in healthy volunteers.

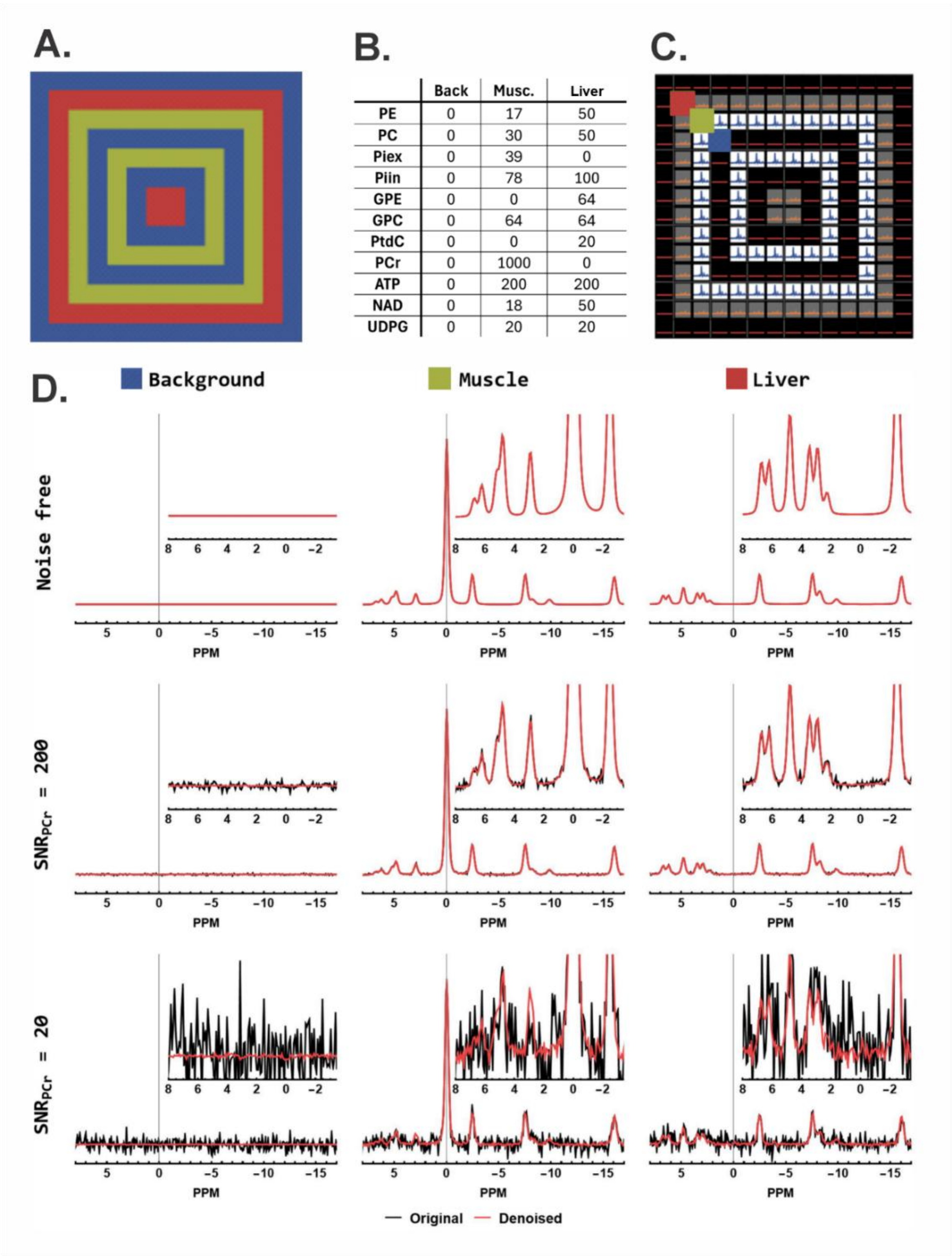

Figure S1. Simulation of denoising performance. Principal-component analysis (PCA)-based denoising was performed on an *in silico* 3D phantom, encompassing three distinct compartments (**A**) representing metabolite content for (**B**) background (**blue**, no signal), skeletal muscle (**green**, high level of phosphocreatine (PCr)), and liver (**red**, no PCr). Spectra are shown for three adjacent voxels (**C**). Spectra (**black**) were simulated at 7 T (bandwidth, 5 kHz; echo time, 0 ms; number of points, 512; line width, 50 Hz) without noise, with a signal-to-noise ratio (SNR) for muscle PCr of 200, and an SNR for muscle PCr of 20 (**D**), while noise power was kept equal between compartments for each set. PCA-based denoising was then applied with a 5 × 5 × 5 kernel identical to the processing of our *in vivo* data. Comparison of original (**black**) and denoised (**red**) spectra demonstrates signal recovery (**D**). Note how noise is effectively reduced in all compartments, while there is essentially no visible mixing or blurring of the high PCr signal from skeletal muscle into the background or liver voxels post-denoising, indicating that spatial signal bleeding between tissue compartments due to PCA-based denoising is negligible.

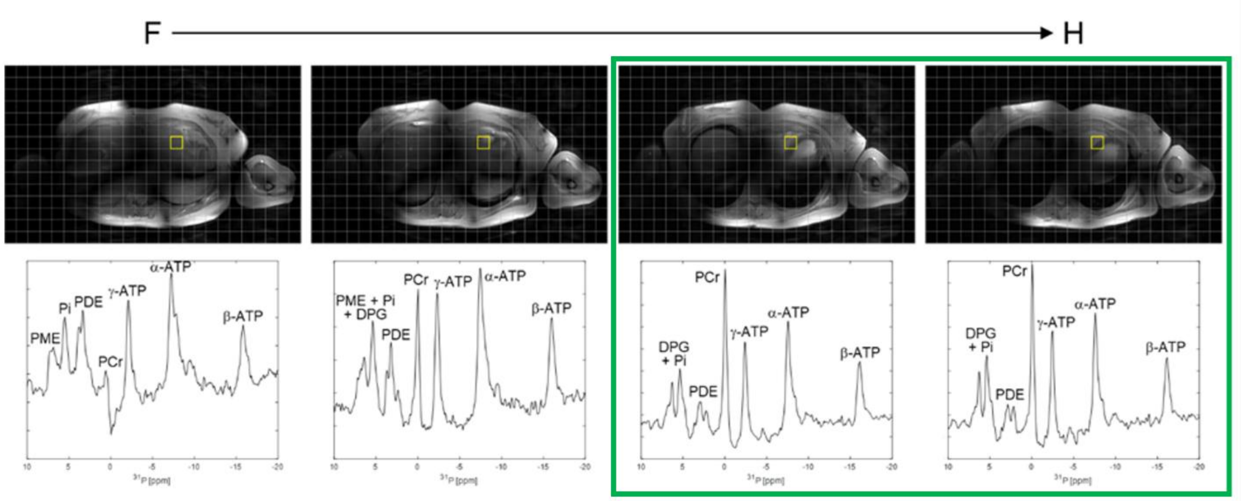

Figure S2. Effect of contamination with signal arising from the liver in voxels adjacent to the heart. Note how only little signal contamination from phosphocreatine (PCr, 0.00 ppm) from the heart or muscle appears in a voxel (**yellow**) selected within the liver in the more caudal slice (F), and that the PCr signal increases in contingent slices in the cranial direction (H). In the two most cranial slices (outlined in **green**), no liver is visible in the subject's left side in the proton MR image, and the ^{31}P-MR spectra are characteristic for myocardial tissue. To minimize any contamination with signal from the liver, heart slices for defining myocardial ^{31}P-MR spectroscopic imaging voxels were selected conservatively: either two slices above (i.e., more cranial) the most cranial slice containing tissue from the left liver lobe in the proton MR image, or one slice above the most caudal slice containing predominantly heart tissue. In this example, the mid-septal voxel for single-voxel analyses was selected in the most cranial slice. Nominal voxel size for ^{31}P-MR spectroscopic imaging, 20 × 20 × 20 mm^3; proton MR image slice thickness/gap, 10/10 mm.

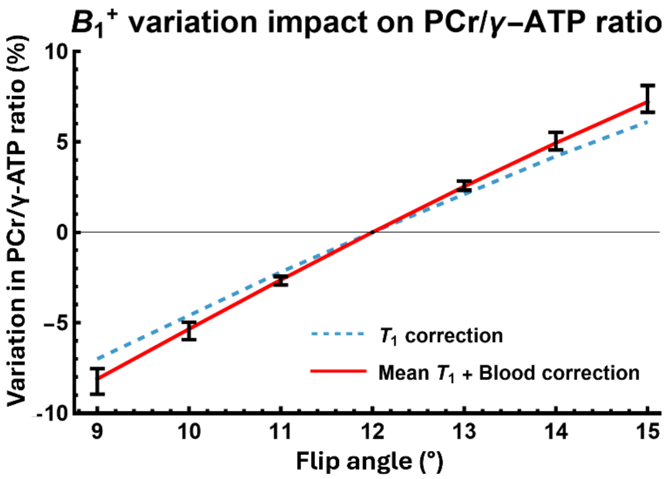

Figure S3. Effect of B_1^+ (i.e., effective flip angle) variation on the phosphocreatine (PCr) to γ-adenosine triphosphate (γ-ATP) ratio, only corrected for partial saturation (**blue** dashed line) or corrected for both partial saturation as well as for blood signal contamination (**red** line). The latter was determined for the full range of voxel blood contributions (based on the 2,3-DPG signal per voxel) as encountered in our study (indicated by the **black** error bars). A ±25% variation in the effective flip angle will lead to mean deviations of only -9% to +8% in PCr/γ-ATP corrected for partial saturation and blood signal contamination.

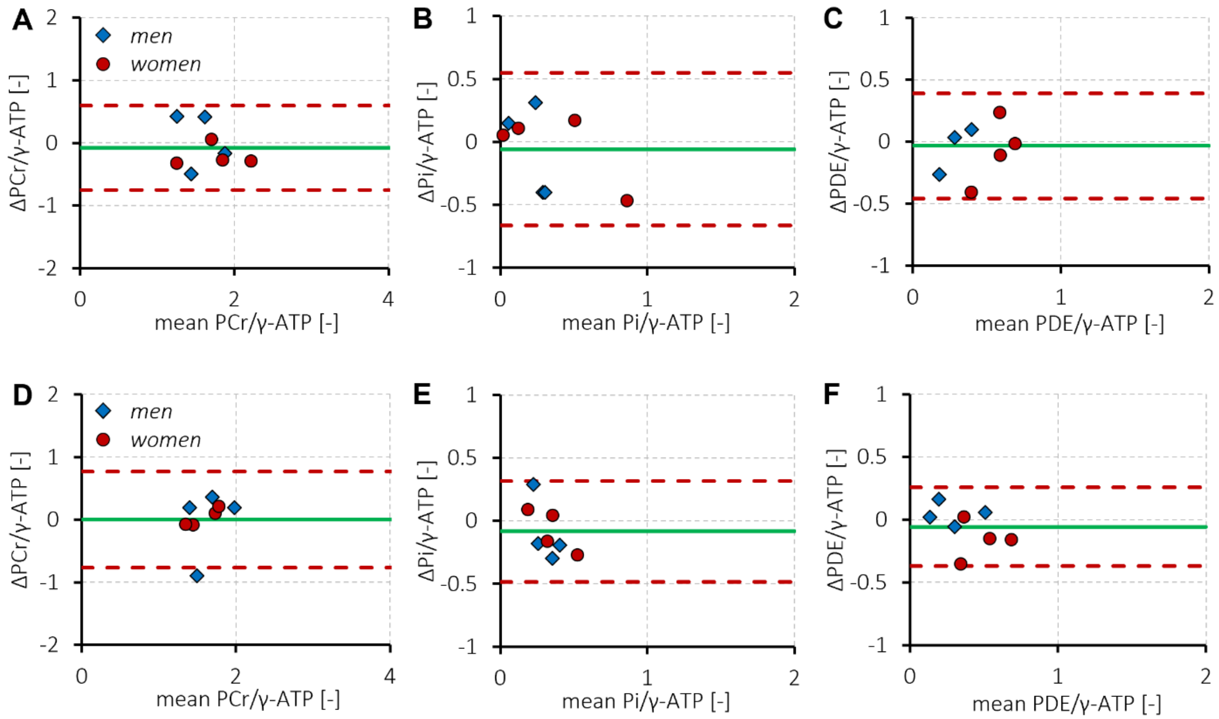

Figure S4. Bland-Altman analysis of the inter-session measurement repeatability for single-voxel septal (**A-C**) and mean myocardial (**D-F**) PCr/γ-ATP (**A, D**), P_i/γ-ATP (**B, E**), and PDE/γ-ATP (**C, F**) measured with ^{31}P-MR spectroscopic imaging at 7 T in men (n = 4; **blue** diamonds) and women (n = 4; **red** circles). Results are based on spectral fits without prior denoising. The dashed **red** lines represent the 95% confidence interval at 1.96 times the standard deviation of the mean difference (**green** solid line) between two consecutive measurements on the same day. The inter-session repeatability coefficient for septal PCr/γ-ATP (**A**) was 41.0% at an overall mean value of 1.65 ± 0.33 with a mean difference between two measurements of -0.08 ± 0.35. The inter-session repeatability for septal P_i/γ-ATP (**B**) was 202.3% at an overall mean value of 0.30 ± 0.27 with a mean difference of -0.06 ± 0.31. For septal PDE/γ-ATP (**C**), the inter-session repeatability coefficient was 111.3% at an overall mean value of 0.38 ± 0.25 and a mean difference of -0.03 ± 0.22. The inter-session repeatability coefficient for mean myocardial PCr/γ-ATP (**D**) was 47.8% at an overall mean value of 1.61 ± 0.22 with a mean difference between two measurements of 0.00 ± 0.39. The inter-session repeatability for mean myocardial P_i/γ-ATP (**E**) was 122.3% at an overall mean value of 0.33 ± 0.11 with a mean difference of -0.08 ± 0.20. For PDE/γ-ATP (**F**), the inter-session repeatability coefficient was 81.4% at an overall mean value of 0.38 ± 0.18 and a mean difference of -0.06 ± 0.16.
